# Prospective diagnostic and prognostic study of copeptin in suspected acute aortic syndromes

**DOI:** 10.1038/s41598-018-35016-z

**Published:** 2018-11-13

**Authors:** Fulvio Morello, Matteo Oddi, Giulia Cavalot, Alice Ianniello, Francesca Giachino, Peiman Nazerian, Stefania Battista, Corrado Magnino, Maria Tizzani, Fabio Settanni, Giulio Mengozzi, Enrico Lupia

**Affiliations:** 1 S.C.U. Medicina d’Urgenza, A.O.U. Città della Salute e della Scienza, Molinette Hospital, Torino, Italy; 2 S.C.U. Medicina Interna 2, A.O.U. Città della Salute e della Scienza, Molinette Hospital, Torino, Italy; 3 S.C. Biochimica Clinica, A.O.U. Città della Salute e della Scienza, Molinette Hospital, Torino, Italy; 4 D.E.A., A.O.U. Careggi, Firenze, Italy

## Abstract

Acute aortic syndromes (AAS) are cardiovascular emergencies with unmet diagnostic needs. Copeptin is released upon stress conditions and is approved for rule-out of myocardial infarction (MI). As MI and AAS share presenting symptoms, stress mechanisms and necessity for rapid diagnosis, copeptin appears as an attractive biomarker also for AAS. We thus performed a diagnostic and observational study in Emergency Department (ED) outpatients. Inclusion criteria were chest/abdominal/back pain, syncope and/or perfusion deficit, plus AAS in differential diagnosis. Blood samples were obtained in the ED. 313 patients were analyzed and 105 (33.5%) were diagnosed with AAS. Median copeptin was 38.91 pmol/L (interquartile range, IQR, 16.33–173.4) in AAS and 7.51 pmol/L (IQR 3.58–15.08) in alternative diagnoses (*P* < 0.001). Copeptin (≥10 pmol/L) had a sensitivity of 80.8% (95% confidence interval, CI, 72.2–87.2) and a specificity of 63.6% (CI 56.9–69.9) for AAS. Within 6 hours, the sensitivity and specificity were 88.7% (CI 79.3–94.2) and 52.4% (CI 42.9–61.8) respectively. Combination with D-dimer did not increase the diagnostic yield. Furthermore, copeptin ≥25 pmol/L predicted mortality in patients with alternative diagnoses but not with AAS. In conclusion, copeptin increases in most patients with AAS within the first hours, but the accuracy of copeptin for diagnosis AAS is suboptimal.

## Introduction

Acute aortic syndromes (AAS) are emergencies affecting 3.5–6 individuals per 100,000 patient-years^1^. They include acute aortic dissection (AAD), intramural aortic hematoma (IMH), spontaneous aortic rupture (SAR) and penetrating aortic ulcer (PAU)^2^. The diagnosis of AAS is extremely challenging because presenting symptoms are unspecific and most diseases in differential diagnosis are more prevalent. A conclusive diagnosis of AAS is based on advanced aortic imaging with computed tomography angiography (CTA)^3^. However, since CTA uses ionizing radiations and may cause contrast-induced nephropathy and anaphylaxis, not all patients with AAS-compatible symptoms should undergo CTA. Therefore, misdiagnosis and over-testing for AAS are still substantial and development of biomarker-assisted diagnostic algorithms constitutes an unmet clinical need^4,5^.

Currently, D-dimer is the only biomarker applicable to suspected AAS in clinical practice^6,7^. However, D-dimer is poorly specific and has limitations, for instance in certain AAS subtypes and in early presenters^7–9^. Therefore, combination of D-dimer with additional biomarkers might be useful. Copeptin is a peptide secreted by the neurohypophysis in response to stress and its levels rise in myocardial infarction and in other critical illnesses^10–13^. A key feature of copeptin is represented by rapid release. Accordingly, combined assay of copeptin and troponin constitutes an early rule-out strategy for myocardial infarction^14^. Myocardial infarction and AAS share stress mechanisms, presenting symptoms, necessity for rapid diagnosis and rule-out. Therefore, copeptin appears as a potentially meaningful biomarker also for suspected AAS, but only preliminary evidence is available^15^. Herein, we specifically evaluated the performance of copeptin, alone and in combination with D-dimer, for diagnostic rule-in and rule-out of AAS in the Emergency Department (ED). We also evaluated copeptin as an early biomarker of mortality, in addition to clinical variables.

## Results

### Patient population

318 ED patients were enrolled and 313 were analyzed (Suppl. Fig. 1). The patient characteristics are presented in Table 1. The presenting symptoms were: anterior chest pain in 223 (71.2%) patients, back pain in 104 (33.2%), abdominal pain in 71 (22.7%), syncope in 34 (10.9%) and suspected perfusion deficit in 54 (17.3%). Conclusive aortic imaging was obtained in 202 (64.5%) patients, while 111 patients were followed-up for diagnostic adjudication. AAS was finally diagnosed in 104 (33.2%) patients: 59 (18.8%) had type A AAD, 20 (6.4%) IMH, 17 (5.4%) type B AAD, 5 (1.6%) SAR and 3 (1%) PAU. 209 (66.8%) patients had an alternative diagnosis: 93 (29.7%) patients had muscle-skeletal pain, 26 (8.3%) gastro-intestinal disease, 19 (6.1%) acute coronary syndrome, 11 (3.5%) uncomplicated aortic aneurysm, 9 (2.9%) pleuritis or pneumonia, 7 (2.2%) syncope, 6 (1.9%) pericarditis, 3 (1%) stroke, 2 (0.6%) pulmonary embolism and 33 (10.5%) other diagnoses.

**Table 1:** Demographic and clinical characteristics of study patients.

Variable	Acute aortic syndromes (n = 104)	Alternative diagnoses (n = 209)	*P*-value
Male gender	75 (72.1%)	131 (62.7%)	0.1
Age (years)	70 (58–80)	59 (50–73)	<0.001
Hypertension	74 (71.4%)	108 (51.7%)	0.001
Diabetes	7 (6.7%)	19 (9.1%)	0.51
Dyslipidemia	7 (6.7%)	51 (24.4%)	<0.001
Smoking	26 (25%)	63 (30.1%)	0.34
Illicit drug use	0 (0%)	1 (0.5%)	0.48
Active cancer	1 (1.4%)	3 (1.8%)	0.81
Coronary artery disease	10 (9.6%)	28 (13.4%)	0.34
Peripheral artery disease	0 (0%)	3 (1.4%)	0.22
Hours from symptom onset	4 (2–8)	7 (2–24)	<0.001
Systolic BP (mm Hg)	140 (105–170)	140 (130–160)	0.3
Diastolic BP (mm Hg)	80 (60–90)	85 (80–90)	0.001
Heart rate (bpm)	75 (65–90)	80 (70–90)	0.18
White blood cells (x10^3^/µl)	11.45 (9.1–14.23)	7.94 (6.5–9.78)	<0.001
Creatinine (mg/dL)	1.07 (0.9–1.28)	0.91 (0.75–1.05)	<0.001
Cardiac troponin T (ng/L)	15 (8–35)	7 (3–11)	<0.001
D-dimer (ng/mL)	7300 (2641–20000)	341 (240–840)	<0.001

Dichotomic variables are represented as n (%). Age, hours from symptom onset and blood test results are presented as median and interquartile range (in brackets). *P*-value was calculated with Pearson’s χ^2^ test for dichotomic variables and with Mann-Whitney *U*-test for continuous variables.

### Copeptin

**Figure 1:**
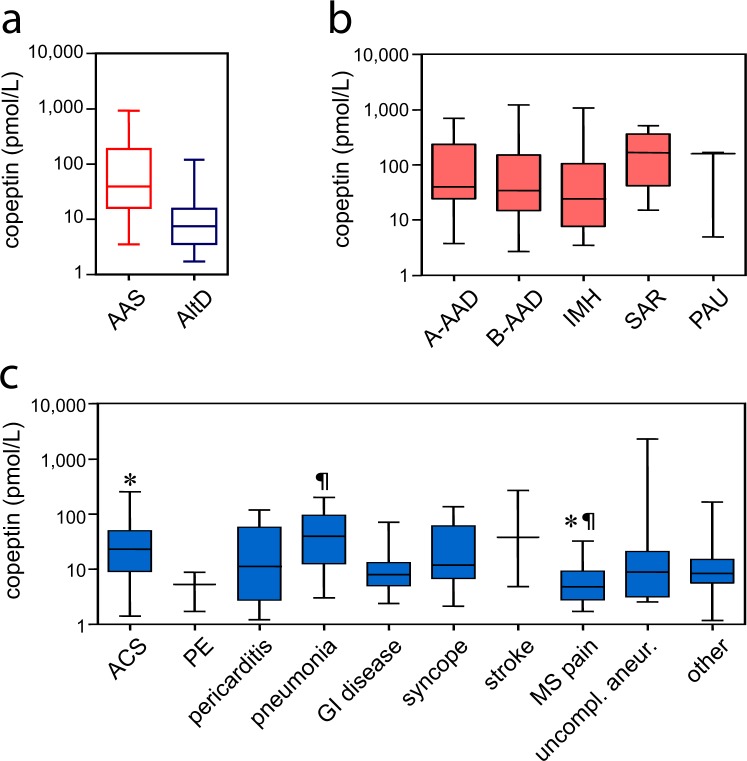
Copeptin levels in study patients. Box and whiskers represent interquartile range, median and 95% confidence interval. On the y-axis, copeptin levels (pmol/L) are reported on a log_10_ scale. (**a**) Copeptin in acute aortic syndromes (AAS) and in alternative diagnoses (AltD). *P* < 0.001. (**b**) Copeptin in subtypes of AAS. A-AAD: type A acute aortic dissection, B-AAD: type B acute aortic dissection, IMH: intramural aortic hematoma, SAR: spontaneous aortic rupture, PAU: penetrating aortic ulcer. (**c**) Copeptin in different alternative diagnoses. *P = 0.001, ^¶^*P* = 0.01. ACS: acute coronary syndrome, PE: pulmonary embolism, GI: gastro-intestinal, MS: muscle-skeletal pain.

**Figure 2:**
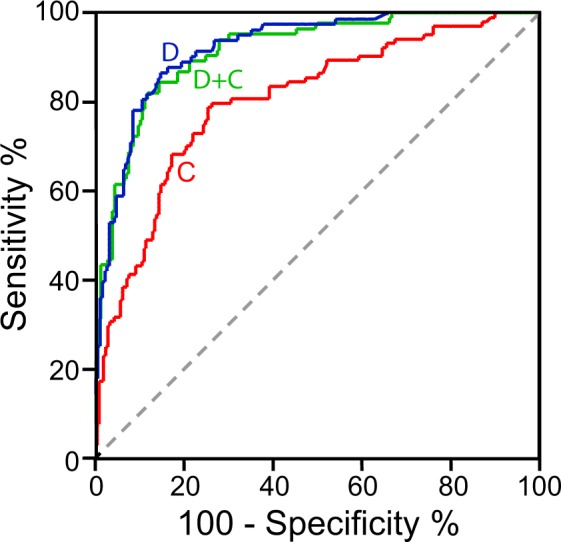
Accuracy of copeptin and D-dimer for diagnosis of acute aortic syndromes (AAS). Receiver operating characteristic curves of copeptin (C, red line), D-dimer (D, blue line) and integration of copeptin and D-dimer (D + C, green line) for diagnosis of AAS.

Median copeptin was 39.33 pmol/L (IQR 16.21–182.5) in AAS and 7.43 pmol/L (IQR 3.54–14.47) in alternative diagnoses (*P* < 0.001; Fig. 1a). Copeptin levels did not statistically differ between subtypes of AAS (Fig. 1b; *P* = 0.46). Amongst alternative diagnoses, higher copeptin levels were found in acute coronary syndromes and in pneumonia/pleuritis (Fig. 1c). In receiver operating characteristic (ROC) curve analysis, the area under the curve (AUC) of copeptin was 0.81 (CI 0.75–0.86; Fig. 2). For AAD or SAR, representing the most severe AAS forms, the AUC was 0.83 (CI 0.77–0.88). For IMH or PAU, the AUC was 0.73 (CI 0.62–0.84). The AUC of copeptin for all AAS subtypes is shown in Suppl. Table 1.

The optimal cutoff of copeptin for diagnosis of AAS was 14 pmol/L. With this cutoff, copeptin had a sensitivity of 78.8% (CI 70.1–85.6), a specificity of 74.6% (CI 68.3–80.1), a positive predictive value (PPV) of 60.7% (CI 52.3–68.6), a negative predictive value (NPV) of 87.6% (CI 82–91.7), a positive likelihood ratio (LR+) of 3.11 (CI 2.41–4) and a negative likelihood ratio (LR−) of 0.28 (CI 0.19–0.41). Using the established cutoff for myocardial infarction (≥10 pmol/L), copeptin had a sensitivity of 80.8% (CI 72.2–87.2), a specificity of 63.6% (CI 56.9–69.9), a PPV of 52.5% (CI 44.8–60.1), a NPV of 86.9% (CI 80.7–91.4), a LR+ of 2.22 (CI 1.81–2.72) and a LR− of 0.3 (CI 0.2–0.45) for AAS. The sensitivity of copeptin for all diagnoses is shown in Suppl. Table 2. Since a high level of sensitivity is required for use in rule-out algorithms of AAS, the established cutoff of 10 pmol/L was applied in further analyses.

Twenty patients with AAS had copeptin <10 pmol/L, corresponding to a false negative rate of 19.2% (CI 12.8–27.8). False negative cases included: 9 type A AAD, 3 type B AAD, 7 IMH and 1 PAU. Copeptin ≥10 pmol/L was statistically associated with presence of syncope, shorter time from symptom onset to sampling and increased levels of creatinine, troponin and D-dimer (Table 2).

**Table 2:** Demographic and clinical characteristics of patients with acute aortic syndrome classified according to the copeptin test result.

Variable	copeptin <10 pmol/L (n = 20)	copeptin ≥10 pmol/L (n = 84)	*P*-value
Male gender	16 (80%)	59 (70.2%)	0.38
Age (years)	69 (54–78)	70 (59–81)	0.39
Hours from symptom onset	12 (2–27)	3 (2–6)	0.018
Anterior chest pain	17 (85%)	50 (59.5%)	0.032
Back pain	10 (50%)	39 (46.4%)	0.77
Abdominal pain	6 (30%)	20 (23.8%)	0.57
Syncope	0 (0%)	15 (17.9%)	0.041
Perfusion deficit	4 (20%)	27 (32.1%)	0.29
Severe pain	13 (65%)	32 (38.1%)	0.029
Pain present in the ED	4 (33.3%)	26 (41.9%)	0.58
Neurologic deficit	1 (5%)	11 (13.1%)	0.31
Hypotension or shock state	1 (5%)	18 (21.4%)	0.09
Systolic blood pressure (mm Hg)	145 (123–160)	140 (100–170)	0.38
Diastolic blood pressure (mm Hg)	88 (73–100)	75 (60–90)	0.07
Heart rate (bpm)	76 (65–100)	75 (65–88)	0.53
White blood cells (×10^3^/µL)	11.5 (8.21–13.27)	11.44 (9.15–14.72)	0.27
Creatinine (mg/dL)	0.9 (0.79–1.1)	1.09 (0.94–1.35)	0.01
Troponin T (ng/L)	7 (5–10)	17 (10–40)	<0.001
D-dimer (ng/mL)	4235 (847–9300)	7870 (2850–20000)	0.033

ED: Emergency Department. Dichotomic variables are represented as n (%). Age, time from symptom onset and blood test results are presented as median and interquartile range (in brackets). *P*-value was calculated with Pearson’s χ^2^ test for dichotomic variables and with Mann-Whitney *U*-test for continuous variables.

### Time from onset

**Figure 3:**
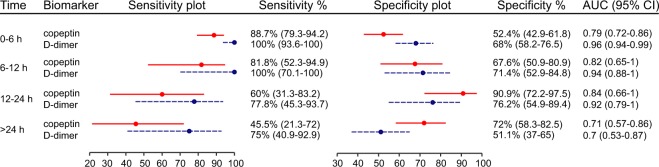
Sensitivity and specificity of copeptin (≥10 pmol/L) and D-dimer (≥500 ng/mL) for diagnosis of acute aortic syndromes (AAS) within different time lags from symptom onset. Right column: area under the curve (AUC) of the corresponding receiver operating characteristic curve for diagnosis of AAS. The number of patients in each group were: 174 for 0–6 hours (h), 45 for 6–12 h, 32 for 12–24 h and 61 for >24 h. Lines represent the 95% confidence intervals (CI) around the central % value (circle).

Time data was available for 312 patients (99.7%). The sensitivity of copeptin was highest when sampling occurred within 6 hours from symptom onset, while the specificity peaked at 12–24 hours (Fig. 3). Within 12 hours from onset (n = 219 patients), the AUC of copeptin for AAS was 0.8 (CI 0.74–0.87). In this time frame, copeptin ≥10 pmol/L had a sensitivity of 87.8% (CI 79–93.2), a specificity of 56.2% (CI 47.8–64.2), a PPV of 54.5% (CI 46.1–62.8), a NPV of 88.5% (CI 80.1–93.6%), a LR+ of 2 (CI 1.63–2.46) and a LR− of 0.22 (CI 0.12–0.4).

### Copeptin plus D-dimer

A D-dimer test result was available for 274 (87.5%) patients. In ROC analysis, D-dimer had an AUC of 0.92 (CI 0.89–0.96; *P* < 0.001 *vs* copeptin, Fig. 2). For AAD or SAR, the AUC of D-dimer was 0.95 (CI 0.93–0.98; *P* < 0.001 *vs* copeptin). For IMH or PAU, the AUC of D-dimer was 0.8 (CI 0.7–0.9; *P* = 0.31 *vs* copeptin). D-dimer ≥500 ng/mL had a sensitivity of 95.2% (CI 88.3–98.1) and a specificity of 65.4% (CI 58.5–71.8) for diagnosis of AAS. The sensitivity and specificity of D-dimer within different time lags from symptom onset are shown in Fig. 3. Within 12 hours from symptom onset, the AUC of D-dimer for diagnosis of AAS was 0.96 (CI 0.94–0.98; *P* < 0.001 *vs* copeptin).

The AUC of the combination of D-dimer and copeptin for diagnosis of AAS (Fig. 2) was 0.92 (CI, 0.88–0.95; *P* = n.s. *vs* D-dimer). Combination of D-dimer and copeptin for diagnostic rule-out of AAS (*i*.*e*. test negative if both D-dimer <500 ng/mL and copeptin <10 pmol/L) led to a sensitivity of 95.2% (CI 88.3–98.1) and a specificity of 46.6% (CI 39.7–53.7). All patients with AAS presenting D-dimer <500 ng/mL also had copeptin <10 pmol/L (Table 3).

**Table 3:** Clinical details of study patients affected by an acute aortic syndrome presenting with both a negative D-dimer and a negative copeptin test result.

Patient n.	Clinical description	Hours from sympt. onset	Type of acute aortic syndrome	D-dimer(ng/mL)	Copeptin(pmol/L)
1	75-y.o. man; history of diabetes mellitus, hypertension, smoking, dyslipidemia	26	IMH	470	4.91
2	59- y.o. man; history of hypertension	18	IMH	270	7.05
3	74- y.o. woman; history of diabetes mellitus, hypertension	27	IMH	313	4.99
4	55- y.o. woman; history of smoking	20	PAU	493	7.5

IMH: intramural aortic hematoma; PAU: penetrating aortic ulcer; y.o.: year-old.

### Mortality

**Figure 4:**
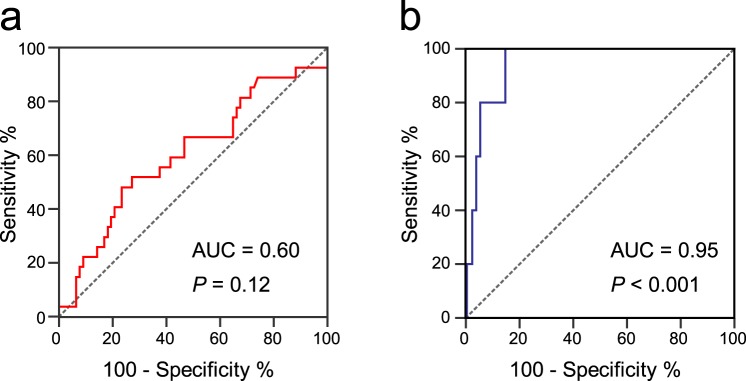
Accuracy of copeptin for mortality prediction. (**a**) Receiver operating characteristic (ROC) curve of copeptin for mortality in patients with acute aortic syndromes (n = 104). (**b**) ROC curve of copeptin for mortality in patients with alternative diagnoses (n = 209). The area under the curve (AUC) and associated *P*-value are reported.

Mortality occurred in 32 (10.2%) patients, including 27 (26%) patients with AAS and 5 (2.4%) patients with alternative diagnoses (*P* < 0.001). ROC analysis showed that copeptin levels predicted mortality in patients with alternative diagnoses (*P* < 0.001), but not in patients with AAS (Fig. 4). In the latter group, the optimal cutoff of copeptin for mortality prediction was 25 pmol/L, providing a sensitivity of 100% (95% CI 47.8–100%), a specificity of 85.3% (95% CI 79.7–89.9%), a PPV of 14.3% (95% CI 4.6–33.3%), a NPV of 100% (0–2.1%), a LR+ of 6.8 (95% CI 4.9–9.5) and a LR− of 0 for mortality. Together with age >70 years, syncope, neurologic deficit, hypotension and D-dimer ≥5000 pmol/L, copeptin ≥25 pmol/L was associated with mortality in univariate analysis (Table 4). None of the selected variables independently predicted mortality in multivariate analysis (data not shown).

**Table 4:** Univariate regression analysis of mortality in study patients with alternative diagnoses to acute aortic syndromes.

Variable	Odds Ratio	95% CI	*P*-value
Age >70 years	24.5	1.3–450	0.03
Female gender	2.4	0.3–22.1	0.43
Hypertension	10.8	0.6–197.6	0.11
Diabetes	0.9	0–16.2	0.92
Smoke	0.6	0–5.2	0.62
Coronary artery disease	0.6	0–10.5	0.7
Syncope	17.6	2.7–113.3	0.003
Severe pain	0.1	0–2.7	0.2
Neurologic deficit	10.7	1.6–70	0.01
Hypotension	67.3	7–650	<0.001
Troponin T ≥ 50 ng/L	5	0.8–31.5	0.09
D-dimer ≥5000 ng/mL*	27.4	2.9–254.6	0.004
Copeptin ≥25 pmol/L	62.9	3.4–1167.5	0.005

CI: confidence interval; *5000 ng/mL represented the optimal cutoff for D-dimer in ROC analysis for mortality prediction in the corresponding subgroup (data not shown).

## Discussion

This is the first study evaluating copeptin as a diagnostic biomarker of AAS. We found that copeptin levels in AAS are higher than reported in myocardial infarction and comparable to those found in other life-threatening conditions such as bleeding, sepsis and critical illness^11,12,14,16–18^. The sensitivity of copeptin for AAS was highest in early presenters to the ED. However, also in this patient group, the accuracy of copeptin appears insufficient for routine application to both rule-in and rule-out diagnostic algorithms of these life-threatening conditions.

Currently, the only universally available biomarker for suspected AAS is D-dimer, which is highly sensitive and poorly specific^6,7^. There are indications that D-dimer may have reduced sensitivity for AAS especially in the first few hours from symptom onset^7^. In the current study, D-dimer largely outperformed copeptin for diagnosis of AAS. The sensitivity of D-dimer was highest within the first 12 hours from symptom onset and within patients with AAD or SAR. All patients with a negative D-dimer had IMH or PAU. Contrary to expectations, combination of copeptin with D-dimer did not provide additional diagnostic accuracy for AAS over D-dimer alone. However, all study patients with AAS presenting a negative D-dimer were sampled more than 12 hours from symptom onset. Further studies focusing on very early presenters and/or on out-of-hospital care are therefore needed to conclusively define whether addition of copeptin to D-dimer may provide any advantage.

In the sub-analysis of patient mortality, copeptin was not an independent predictor of death in patients with AAS. Instead, copeptin was associated with mortality in patients with alternative diagnoses. This is in line with previous findings that copeptin has prognostic implications in other acute conditions such as decompensated heart failure, syncope and sepsis, with higher levels of copeptin defining increased risk of death^10–13^. Taken together, the present results enforce the notion that in the ED, a high copeptin level should be regarded as a red flag for disease severity^15^. This may have clinical implications while evaluating dismissal *vs* hospital admission and the best intensity of care setting for admitted patients (*i*.*e*. general ward *vs* intensive care unit). However, current mortality data refers to a clinically heterogeneous patient population, while prognostic markers should ideally be applied to well-defined etiological entities.

The present study has limitations. First, convenience sampling likely introduced some degree of patient selection bias. Demographic and clinical characteristics of study patients were similar to those reported in previous studies on AAS by this and other groups, but the prevalence of AAS as a final diagnosis was higher than previously reported^7,19^. For the same reason, the present cohort is not suitable for biomarker evaluation in the context of pre-test probability assessment. Second, the present study was underpowered for the sub-analysis of early ED presenters with a negative D-dimer test result. Finally, since mortality was not the primary aim of the study, the study was also underpowered for subgroup analysis of patient mortality, which therefore needs further scrutiny.

In conclusion, the accuracy of copeptin is suboptimal for use in diagnostic algorithms of AAS. In particular, a negative copeptin test should not be used to conclusively rule-out AAS without CTA. After exclusion of AAS, presence of high copeptin levels defines a heterogeneous group of acute patients at higher risk of death.

## Methods

### Study design

We performed a prospective diagnostic accuracy and observational study in a convenience sample of outpatients from a large urban ED. The local Human Ethics Committee (Comitato Etico Interaziendale A.O.U. Città della Salute e della Scienza di Torino) approved the study and written informed consent was obtained from participants. The study protocol conformed to the ethical guidelines (Declaration of Helsinki, 1975).

From January 2014 to July 2017, ED outpatients aged >18 years were eligible if they experienced ≥1 of the following symptoms dating ≤14 days: chest pain, abdominal pain, back pain, syncope, signs or symptoms of perfusion deficit. Patients were included only if AAS was considered in the differential diagnosis by the attending physician. Exclusion criteria were trauma, unwillingness or inadequacy to participate in follow-up and missing copeptin measurement.

The study used convenience sampling for practical reasons. Clinical assessment was performed 24 hours/day and 7 days/week, while immediate plasma extraction, aliquoting and storage was possible for an average of 12 hours/day and 5 days/week. The expected enrolment rate was therefore ≈1 in 3 clinically eligible patients.

### Medical workup

The ED visit included physical exam, ECG and blood sampling. The diagnostic workup of patients reflected current guidelines and was independent of the present study^3^. The standard imaging method allowing conclusive diagnosis of AAS was chest and abdomen contrast-enhanced CTA. All patients for whom conclusive diagnostic data was not obtained entered a 14-day follow-up to allow case adjudication^7^.

### Blood tests

Patients underwent venipuncture in the ED. Blood samples were immediately sent to the laboratory. The laboratory technicians were unaware of clinical data. The white blood cell count was performed on samples collected in EDTA-containing tubes with an automatic counter (XE-2100 Hematology Analyzer, Sysmex Corporation, Japan). Creatinine and high sensitive troponin T were measured with an automated chemistry analyzer (Cobas C8000, Roche Diagnostics GmbH, Mannheim, Germany). D-dimer levels were measured with the automated STA LIATEST™ D-DI assay, based on latex agglutination (Diagnostica Stago for Roche Diagnostics GmbH, Mannheim, Germany).

### Copeptin

Plasma was rapidly obtained from blood by centrifugation of an EDTA-containing tube at 4000 rpm for 5 minutes and a plasma aliquot was immediately frozen and stored at −80 °C in the laboratory until further assay. Copeptin concentrations were subsequently determined using the BRAHAMS KRYPTOR compact PLUS automated method. This is a quantitative test allowing the assessment of copeptin pro-AVP concentrations in human plasma (EDTA or heparin) by time-resolved amplified cryptate emission (TRACE) technique, which measures the signal that is emitted from an immunocomplex with time delay. The assay has a functional sensitivity of 0.9 pmol/L. Imprecision evaluation tests yielded a within-run variation of less than 7% and a between-run variation of less than 12% on a wide range of values.

### Case adjudication

Case adjudication was performed by two expert physicians who independently reviewed the data obtained during the ED visit and follow-up, blinded to copeptin levels. Case adjudication was dichotomic. In case of discordance, the case was adjudicated after discussion. Mortality was checked in the index visit records, in subsequent ED records and in all hospital admission events that followed the index visit.

### Outcomes and power

The primary outcome was the diagnostic accuracy of copeptin for diagnosis of AAS. Secondly, we evaluated the performance of copeptin in association with D-dimer for diagnosis of AAS and mortality associated with copeptin levels.

The present study was powered to test the null hypothesis that plasma copeptin is comparable in AAS and in alternative diagnoses. Assuming that copeptin levels would be 15 ± 30 pmol/L in patients without AAS, an enrolment ratio of 2, a type I error of 0.05 and type II error of 0.1, we estimated that at least 224 patients needed to be included.

### Statistical analysis

Characteristics are reported with median plus interquartile range (IQR) for continuous variables and proportions plus 95% confidence interval (CI) for categorical variables. Statistical differences were compared with non-parametric Mann-Whitney *U*-test, Kruskal-Wallis with *post*-*hoc* Dunn’s multiple comparison test for independent samples or with Pearson’s χ^2^ test.

The diagnostic performance of copeptin was assessed with receiver operating characteristic (ROC) analysis. The area under the curve (AUC) was estimated *per* Hanley and McNeil and AUC comparison was performed *per* DeLong. The Youden’s index was calculated as: sensitivity + specificity −1. The maximum value of the index was used as a criterion for selecting the optimal cut-off point. The following variables were calculated: sensitivity, specificity, PPV, NPV, LR+ and LR−.

Survival analysis was performed by constructing Kaplan Meier curves and by log-rank testing. To identify the variables associated with mortality, contingency tables were constructed, and odds ratios were computed. Pearson’s χ^2^ test was applied. All variables tested in univariate analysis were introduced in the multivariable binary logistic regression analysis. Statistical computations were conducted with SPSS software ver. 20 (IBM) and R ver. 3.0.2. *P*-values were considered significant if <0.05.

## Electronic supplementary material

Supplementary Material

## Data Availability

The datasets generated during and analyzed during the current study are available from the corresponding author on reasonable request.
