# Supplementary material for: Prospective diagnostic and prognostic study of copeptin in suspected acute aortic syndromes
Source: Sci Rep. 2018 Nov 13;8:16713. doi: 10.1038/s41598-018-35016-z (PMC6233166; doi:10.1038/s41598-018-35016-z)
Supplement: Supplementary file 1 — Supplementary Material [file 41598_2018_35016_MOESM1_ESM.pdf]

Prospective diagnostic and prognostic study of copeptin in suspected acute aortic syndromes

Fulvio Morello[1*], Matteo Oddi[1], Giulia Cavalot[2], Alice Ianniello[3], Francesca Giachino[1], Peiman Nazerian[4], Stefania Battista[1], Corrado Magnino[1], Maria Tizzani[1], Fabio Settanni[3], Giulio Mengozzi[3], Enrico Lupia[1].

[1]S.C.U. Medicina d'Urgenza, A.O.U. Città della Salute e della Scienza, Molinette Hospital, Torino, Italy.

[2]S.C.U. Medicina Interna 2, A.O.U. Città della Salute e della Scienza, Molinette Hospital, Torino, Italy.

[3]S.C. Biochimica Clinica, A.O.U. Città della Salute e della Scienza, Molinette Hospital, Torino, Italy.

[4]D.E.A., A.O.U. Careggi, Firenze, Italy.

SUPPLEMENTAL MATERIAL

SUPPLEMENTAL TABLES

Supplemental table 1. Diagnostic accuracy of copeptin and D-dimer for different subtypes of acute aortic syndrome.

Subtype	ROC curve AUC (95% CI)		***P*-value**
	**copeptin**	**D-dimer**	
A-AAD	0.825 (0.763-0.888)	0.961 (0.939-0.983)	<0.001
B-AAD	0.8 (0.691-0.909)	0.933 (0.891-0.974)	0.035
IMH	0.726 (0.611-0.842)	0.808 (0.696-0.92)	0.17
SAR	0.921 (0.836-1)	0.946 (0.908-0.983)	0.69
PAU	0.762 (0.426-1)	0.758 (0.581-0.936)	0.95

A-AAD: type A acute aortic dissection, B-AAD: type B acute aortic dissection, IMH: intramural aortic hematoma, SAR: spontaneous aortic rupture, PAU: penetrating aortic ulcer. *P*-value was calculated according to DeLong.

Supplemental table 2. Sensitivity of copeptin for adjudicated diagnoses.

			sensitivity % (95% CI)	
**Diagnosis**		**n**	**copeptin ≥10 pmol/L**	**copeptin ≥14 pmol/L**
Acute aortic syndromes	A-AAD	59	84.8% (73.5-91.8)	83.1% (71-91.6)
	B-AAD	20	65% (43.3-81.9)	55% (31.5-76.9)
	IMH	17	82.4% (59-93.8)	76.5% (50.1-93.2)
	SAR	5	100% (56.6-100)	100% (47.8-100)
	PAU	3	66.7% (20.8-93.9)	66,7 (9.4-99.2)
Alternative diagnoses	MS pain	93	21.5% (14.4-30.9)	11.8% (6.7-20)
	GI disease	26	26.9% (13.7-46.1)	23.1% (11-42.1)
	ACS	19	73.7% (51.2-88.2)	57.9% (36.3-76.9)
	Uncompl. aneurism	11	36.4% (15.2-64.6)	27.3% (9.7-56.6)
	Pleuritis/pneumonia	9	77.8% (45.3-93.7)	77.8% (45.3-93.7)
	Syncope	7	71.4% (35.9-91.8)	42.9% (15.8-75)
	Pericarditis	6	50% (18.8-81.2)	33.3% (9.7-70)
	Stroke	3	66.7% (20.8-93.9)	66.7% (20.8-93.9)
	PE	2	0% (0-65.8)	0% (0-65.8)
	Other diagnoses	33	42.4% (27.2-59.2)	24.2% (12.8-41)

95% CI: 95% confidence interval. A-AAD: type A acute aortic dissection, B-AAD: type B acute aortic dissection, IMH: intramural aortic hematoma, SAR: spontaneous aortic rupture, PAU: penetrating aortic ulcer. ACS: acute coronary syndrome, GI: gastro-intestinal, MS: muscle-skeletal pain, PE: pulmonary embolism.

SUPPLEMENTAL FIGURE 1

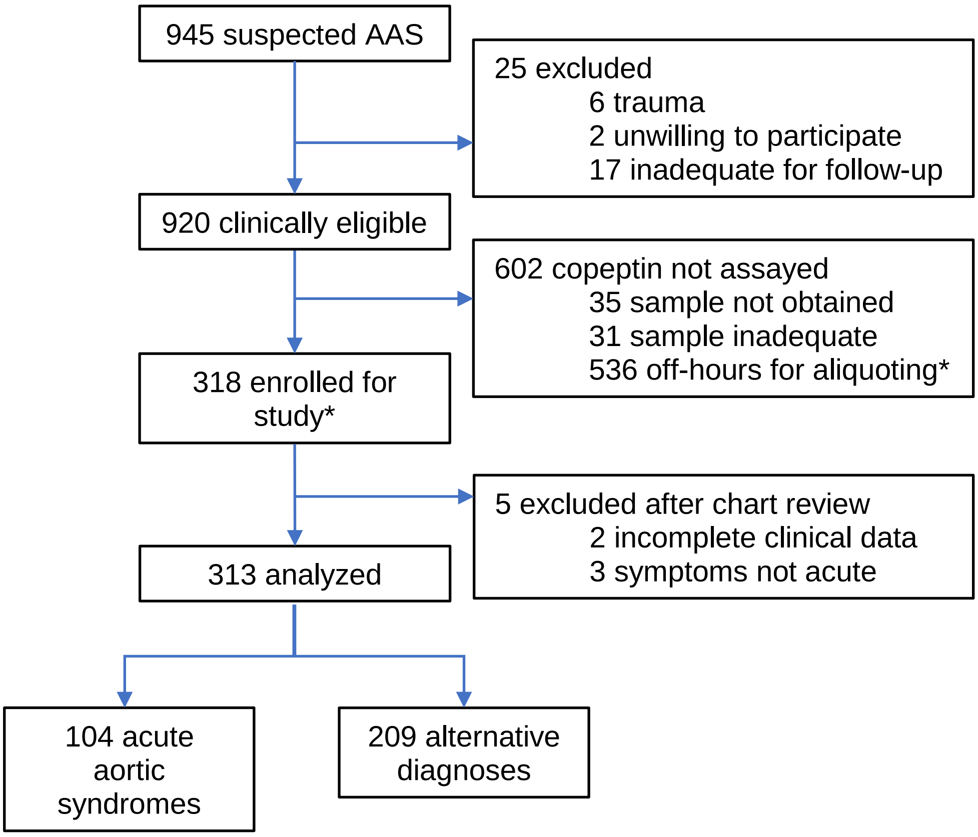

Supplemental figure 1. Study flowchart. *318 clinically eligible patients were enrolled and subjected to copeptin assay. See Method section for convenience sampling details.
